# Effects of Impulsivity on Competitive Anxiety in Female Athletes: The Mediating Role of Mindfulness Trait

**DOI:** 10.3390/ijerph19063223

**Published:** 2022-03-09

**Authors:** Lara Terres-Barcala, Natalia Albaladejo-Blázquez, Raquel Aparicio-Ugarriza, Nicolás Ruiz-Robledillo, Ana Zaragoza-Martí, Rosario Ferrer-Cascales

**Affiliations:** 1 Department of Health Psychology, University of Alicante, 03690 Alicante, Spain; l.terresbarcala@gmail.com (L.T.-B.); nicolas.ruiz@ua.es (N.R.-R.); rosario.ferrer@ua.es (R.F.-C.); 2 Imfine Research Group, Department of Health and Human Performance, Universidad Politécnica de Madrid, 28040 Madrid, Spain; apariciougarriza.raquel@gmail.com; 3 Department of Nursing, Faculty of Health Science, University of Alicante, 03690 Alicante, Spain; ana.zaragoza@ua.es

**Keywords:** sport players, physical activity, anxiety, mindfulness, impulsivity, female athletes

## Abstract

It has been demonstrated that athletes in competitive sports suffer from high levels of competitive anxiety, especially in the case of females. In this sense, it is necessary to identify possible risk and protective factors of those athletes in this collective who suffer from this type of anxiety. However, few studies analyze the relationship between Physical Activity (PA) and anxiety, identifying the possible mediation effect of trait variables such as impulsivity and mindfulness in female athletes. Hence, the aims of this study were: to determine differences between PA levels with anxiety, mindfulness, and impulsiveness; to identify the predictive value of sociodemographic factors and physical activity, impulsivity, and mindfulness on anxiety factors; and to analyze the possible mediating effects of mindfulness on the relationship between impulsivity and anxiety. A total of 242 female athletes underwent an assessment of physical activity, anxiety, mindfulness traits, and impulsivity using validated questionnaires. Data were analyzed according to (1) individual or collective sport, and (2) PA levels according to energy expended (METs min/day). Participants were grouped by light, moderate, and vigorous PA levels. There were 30.5% elite athletes and 73.2% collective sports athletes. Mean age was 22.1 years and mean light, moderate, and vigorous PA were 86.1 ± 136.2, 114.4 ± 159.8, and 370.1 ± 336.3 METs min/day, respectively. Those athletes performing vigorous PA exhibited lower levels of impulsiveness and higher mindfulness traits. As expected, the mindfulness trait was a mediating factor in the relationship between impulsiveness and each factor of competitive anxiety (cognitive, somatic, and self-efficacy). Female athletes could suffer competitive anxiety, especially those who present higher levels of impulsivity. However, higher levels of mindfulness traits seem to be a protective factor in the effects of impulsivity on anxiety in this population and have demonstrated to be significant mediators in this association. Further studies are needed with other female athletes to replicate these results and to determine the specific protective mechanisms of mindfulness traits in preventing competitive anxiety.

## 1. Introduction

It has been demonstrated that sports athletes need to strike a balance between high physical and cognitive performance and self-regulation [1]. Studies show that female and younger athletes presented a greater risk of having anxiety levels than male and older athletes [2,3,4]. However, few studies analyze the association between these factors in female athletes. 

Physical Activity (PA) in elite sports practice, defined as the highest level of competition or high-performance sport, showed that the higher the frequency of sports practice, the greater the benefits on health, well-being, and quality of life perceived by the subject [5,6,7]. However, recent studies display contradicting results as to the benefits of intense PA in competitive sports, given that the practice of elite sports, understood as the maximum exponent of competitiveness in sports, is not always synonymous with well-being and health [8,9,10,11,12,13]. In this sense, competitive sports practice without adequate recovery and a load of both physical and mental training [14] could result in overtraining, physical and mental fatigue, and, therefore, be detrimental to health and negatively impact sports performance, given its association with higher levels of depression, stress, and anxiety [3,10,15,16,17,18,19,20].

Other variables such as impulsivity can act as a risk factor in athletes [1,21,22,23,24], negatively impacting female athletes’ performance [25]. Impulsivity is defined as a predisposition to take quick and unreflective actions in response to internal and/or external stimuli despite the negative consequences. Negative actions could have consequences both for oneself and/or for other individuals [26]. There are three types of impulsivities proposed by Patton et al. [27]: motor (acting without thinking), attentional (lack of focus on the task and hand), and non-planning (focus on the present without accounting for the consequences of the future outcomes). In studies with elite athletes of both genders, greater impulsivity is observed in female athletes than in male athletes [28], though other studies have shown opposite results in not finding significant differences in impulsivity levels according to gender [29]. In relation to variables such as anxiety, high levels of sensitivity to anxiety show a positive correlation with impulsivity in athletes [30,31]. In this regard, it is the most successful athletes who have better emotional control and self-confidence in stressful situations [32,33], and the greater the athlete’s experience in sports, the fewer their impulsive decisions [34]. Although no differences are observed between types of sport [34,35], it has been reported that low levels of impulsivity are related to better tactical behavior in team sports [36,37] and that high rates of impulsivity affect motor performance and decision-making in female athletes [25]. 

Against this background, competitive anxiety is one of the most prevalent types of anxiety in this context. Competitive anxiety is defined as a negative emotional reaction that results from the discrepancy between the environmental demand and the athlete’s response capacity. Furthermore, a differentiation is made between state anxiety and trait anxiety, alluding to its transitory or stable nature, respectively [17,18,38,39]. The Multidimensional Theory of Competitive Anxiety is an approach from a perspective with three main factors identified: cognitive, somatic, and self-confidence anxiety [17]. According to this theory, cognitive anxiety refers to negative thoughts, expectations, and/or self-verbalizations concerning the competitive event, whereas somatic anxiety indicates the affective and physiological elements that directly affect the central nervous system. Self-confidence, in turn, is related to the level of confidence and perceived readiness to compete [17,24,38,39,40,41]. First, the cognitive dimension of anxiety refers to repetitive thoughts about being able or unable to achieve the expected result, the difficulty, and the inability to maintain attention and concentration. Second, the somatic dimension of anxiety refers to the perceived activation of the autonomic nervous system (increased heart rate, palpitations, increased sweating rate, increased respiratory rate, muscle tension, and stomach discomfort) [17,38]. A third dimension is self-confidence or the belief or degree of assurance that individuals have about their ability to attain success in a sport and about their ability to exert control over the environment. Although it is not a direct measure of anxiety, its absence may lead an athlete to experience cognitive anxiety with greater intensity [17,39,40,41].

A review has pointed out that there are several explanation mechanisms for the link between impulsiveness and anxiety [42]. As indicated in this review, some previous research has found significant associations between impulsiveness and anxiety disorders [43,44]. Hence, there is a high comorbidity between anxiety and impulse control disorders [45]. As proposed by the authors, probably, patients with higher levels of anxiety could tend to develop impulsive behaviors when suffering from negative internal emotional states, such as negative affect or uncertainty [42]. In this sense, impulsivity has been demonstrated to be a significant predictor of the severity of symptoms in generalized anxiety disorder [46]. These results have been replicated when the association between impulsivity and anxiety symptoms has been tested in other disorders, such as social anxiety disorder [43]. Beyond anxiety disorders, impulsivity has been demonstrated to be a significant predictor of anxiety dimensions. In this regard, in a study conducted with undergraduate students, components of impulsiveness such as negative urgency and a lack of perseverance significantly predicted higher levels of worry [47]. This fact is especially important in athletes, in which emotion-related impulsiveness, such as negative urgency, could be especially important. In this regard, it has been recently found that impulsiveness in highly stressful situations could lead to anxiety reactions more easily [48]. Hence, individuals with higher levels of emotion-related impulsivity could be at high risk of developing higher levels of internalizing symptoms such as anxiety, due to their proneness to worry in ambiguous situations. Other facets of impulsiveness, such as the motor component, have also been directly associated with anxiety [49]. Based on this previous research, it seems that impulsiveness could be a risk factor for the development of anxiety symptomatology, but new studies are needed to clarify this relationship in specific populations, such as athletes.

Different studies indicate that in the face of negative consequences such as stress and anxiety, there are variables such as mindfulness that act as a protective factor in diverse contexts, such as competitive sports [50,51,52]. Mindfulness means the acceptance and awareness of thoughts, emotions, physical senses, and external experiences at the present time without judging and trying to control and suppress them [12]. Moreover, trait mindfulness is distinct from state mindfulness, which describes the non-judgmental present-focused awareness experienced in any given moment [53]. In this sense, we found studies on elite female athletes where mindfulness programs were shown to reduce negative thoughts and promote sports performance [12,33,54,55,56,57,58]. Specifically, these programs can be effective in reducing precompetitive anxiety [59], and women athletes, in comparison to men, report higher levels of mindfulness [60]. Nowadays, studies stress the importance of mindfulness programs in elite female athletes in both team sports [61] and individual sports [59,62]. 

At the international level, most studies have focused on evaluating physical and sports activity in male elite athletes, as well as its relationship with different psychological variables such as stress and anxiety [15,16,63,64]. However, there are few studies carried out with female elite athletes. In this sense, it is important to highlight that several studies have revealed gender differences in the association between elite sports practice and psychological well-being [65,66]. In the case of men, intense and long-lasting PA has a protective role against anxiety states [40,64]; however, when PA is associated with sports or elite performance, anxiety could be a risk factor for these athletes [12,41,63,67]. In elite female athletes, higher levels of precompetitive anxiety are reported compared to male athletes [65,68,69]; however, this area of research remains largely unexplored. As to the type of sport, male athletes in individual sports versus team sports show higher levels of cognitive anxiety and lower tolerance to errors [70,71]. It is important to highlight that several studies with men and women have shown that the group of female athletes in elite individual sports obtain the highest scores in sports anxiety [69,72] with no previous studies analyzing moderate and high PA and its relationship with anxiety.

In this sense, despite the acknowledgement of some progress, there is evidence of the need to analyze PA levels and their relationship with variables such as competitive anxiety, as well as the influence of trait variables, such as mindfulness and impulsivity in this relationship, which may play a mediating role in this association. The differences between individual and group sports in female competitive athletes also deserve attention.

Therefore, the aims of this study were to (1) determine differences between PA levels with anxiety, mindfulness, and impulsiveness; (2) identify the predictive value of sociodemographic factors and physical activity, impulsivity, and mindfulness on anxiety factors; (3) and analyze the possible mediating effects of mindfulness on the relationship between impulsivity and anxiety.

## 2. Materials and Methods

### 2.1. Study Design and Participants

This research was a cross-sectional descriptive study. The sample consisted of 242 female individual and team athletes with a mean age of 22.01 (SD = 5.02). The sample was selected based on intentional non-probabilistic sampling, in accordance with the following inclusion criteria: (a) participants were women over 15 years of age, (b) national, international, and/or university level, individual or team female athletes, (c) competing in Spain, (d) having a federative license in 2018–2019, and (e) who had signed the written informed consent.

Table 1 describes the 242 participants of the study sample. The mean age was 22.1 years and 73.2% of the participants played in a collective sport. A total of 30.5% were elite athletes and the total energy expended was 561 min per day.

### 2.2. Measures

A data collection questionnaire was prepared including sociodemographic and anthropometric data (age, height, weight, and educational level); and information related to sports career (sports years, competition level, or type of sport). PA was measured using the International Physical Activity Questionnaire (IPAQ) [73] adapted to the Spanish IPAQ short version by Román et al. [74] This 7-item questionnaire records the amount of physical activity in the last seven days. Minutes at each intensity level exceeding 180 min per day were truncated to 180 min/day to avoid extreme outliers. Nevertheless, the maximum for moderate to vigorous PA (MVPA) was established at 360 min/day (2520 min/week), as a sum of moderate PA (180 min/day) and vigorous PA (180 min/day). Moreover, total PA as the sum of low (180 min/day), medium (180 min/day), and vigorous (180 min/day) scored a total of 540 min/day (3780 min/week). The score was calculated according to established methods, quantifying PA as metabolic equivalent (MET) min/day available on the IPAQ website (www.ipaq.ki.se, accessed on 29 November 2021). PA level was quantified as metabolic equivalent METs hours/week by multiplying the METs, duration, and frequency of activities from exercise; data were presented as METs min/day. The value of METs for each PA was 3.3. METs for light exercise, 4 METs for moderate exercise, and 8 METs for vigorous exercise according to the IPAQ’s guidelines for data processing and analysis. Participants were also categorized as low, moderate, and vigorous PA levels by METs (min/week). Sitting time was also included in this study using IPAQ (min/day). This variable was measured in minutes per day. The IPAQ short version has a metric of 0.76 for validity and reliability.

For the assessment of pre-competition anxiety, the Competitive State Anxiety Inventory *2*, CSAI-2 [75], modified to Spanish by Andrade, Lois, and Arce [76], was used. The questionnaire comprises 27 items that assess three different variables: cognitive anxiety, self-confidence, and somatic anxiety (nine items for each variable). Elements are answered on a Likert scale from 1 (nothing) to 4 (a lot). The total score is a maximum of 36 and a minimum of 9 points. The internal consistency of the coefficients is between 0.79 and 0.82. 

Mindful Attention Awareness Scale, MAAS [77], adapted to the Spanish version by Soler et al. [78], was used to evaluate individual differences in the frequency of mindfulness over a period of time. It consists of 15 items, each an affirmation expressed as a declarative sentence. Subjects gauge how frequently they have experienced a given situation using a 6-point Likert scale. Higher scores indicate greater mindfulness traits. It demonstrates good psychometric properties (Cronbach’s alpha = 0.89). Internal consistency (Cronbach’s alpha) for this study was good (α = 0.88).

To evaluate the personality/behavioral construct of impulsiveness, the Barratt Impulsiveness Scale-11 (BIS-11) [27], modified to the Spanish version by Salvo and Castro [79] was run. It contains 30 items that are self-rated on a scale of 1 to 4: (1) rarely/never, (2) occasionally, (3) often, and (4) almost always. It assesses the three main dimensions of impulsive behavior: attentional (a lack of focus on the ongoing task), motor (acting without thinking), and non-planning impulsivity (orientation to the present rather than to the future). The total score ranges from 30 to 120 and higher scores indicate greater impulsivity. It has reported internal consistency (Cronbach’s alpha = 0.77). Internal consistency (Cronbach’s alpha) for this study was good (α = 0.77).

### 2.3. Procedure

The sample was recruited between 30 October 2018 and 30 March 2019. Two informative meetings were held at different sports institutions (sports clubs, sports associations, training centers, services university sports) to inform the participants in the study about the study’s aims. After obtaining permission, firstly, an information meeting with female athletes was held to explain the aims of the study. Secondly, each female athlete was given an information sheet and informed consent. The assessment was performed in groups during a competition or training. The evaluation lasted around 35 min, and all the female athletes received similar guidelines. The evaluation was performed by a team of experienced experts in this type of research. Informed consent was also obtained from legal tutors and parents of participants under 18 years of age, in accordance with Royal Decree 1720/2007 on the Protection of Personal Data. All the personal data obtained in this study were processed in accordance with Organic Law 3/5 December 2018, on Protection of Personal Data and Guarantee of Digital Rights as stipulated in Regulation (EU) 2016/679 of the European Parliament and of the Council, of 27 April 2016 on Data Protection (GDPR). The study was approved by the Ethics Committee of the University of Alicante (UA-2020-11-20). 

### 2.4. Data Analysis

Different descriptive analyses (mean ± SD) were carried out. The Kolmogorov–Smirnov test was run to assess compliance with normality once all the information on PA was transformed into METs/day. All the variables were normally distributed except low, moderate, and vigorous PA, MVPA, and total PA. Regarding psychological questionnaires, only social anxiety and cognitive impulsivity showed non-normal distribution. Thus, differences by groups were performed using Kruskal–Wallis and ANOVA for non-normal and normal variables, respectively.

Standardization and magnitude-based inferences with 95% confidence limits (CL) were used to assess differences between groups following the Cohen’s D (d = 0.20 small effect, d = 0.50 medium effect, and d = 0.80 large) effect. In our results, Cohen’s D ranges from 0.38 through to 0.54. 

Hierarchical regression analyses were performed to analyze the role of sociodemographic and physical activity, impulsivity, and mindfulness trait variables as predictors of anxiety factors. Model 1 included only the sociodemographic characteristics and physical activity of the sample. Model 2 included sociodemographic, physical activity, and impulsiveness factors. Model 3 included sociodemographic, PA, impulsiveness, and mindfulness traits. 

For the mediation analyses, the PROCESS macro by Hayes was used [80]. This macro is a path analysis modelling tool used to estimate direct and indirect effects in mediation models. It is an empirical bias-corrected bootstrapping procedure that serves to approximate confidence intervals from the repeated resampling of the observed data. A mediation effect is significant only when the 95% confidence interval does not include zero. In the present study, the data were resampled 10,000 times, as recommended by Hayes [59]. In small samples, bootstrapping has been demonstrated to be the most effective and powerful method to test indirect effects compared to other traditional methods, such as linear regression or the Sobel test.

Analyses were performed using SPSS version 22.0 (SPSS, Inc., Chicago, IL, USA). The level of significance was set at *p* < 0.05.

## 3. Results

### 3.1. Clinical Characteristics

Participants self-reported scores of 2.5, 4.1, and 67.2 in anxiety, impulsivity, and mindfulness (Table 2).

### 3.2. Differences between PA Categories with Anxiety, Mindfulness, and Impulsivity 

Table 3 presents the comparison between light-moderate PA and vigorous PA with sitting time, anxiety, mindfulness, and impulsivity. Individuals who performed light-moderate PA, showed significant higher levels of total impulsiveness (69.42, *p* < 0.05), attentional impulsiveness (20.22, *p* = 0.034), and motor impulsiveness (24.16, *p* = 0.001) compared to vigorous PA group. Significant differences were also observed between light-moderate PA and vigorous PA with mindfulness (3.91 vs. 4.23, respectively) (*p* = 0.004).

### 3.3. Predictive Value of Sociodemographic and Physical Activity, Impulsivity and Mindfulness on Anxiety Factors

Table 4 shows the regression models between sociodemographic and PA, impulsivity, and mindfulness trait variables as predictors of anxiety factors. PA levels were only a significant predictor of the component of self-efficacy of anxiety. For the remaining predictor variables, although impulsiveness was a significant predictor for each component of competitive anxiety, impulsiveness was not significant when the mindfulness trait was introduced in the regression model.

### 3.4. Mediation Analyses

Mediation analyses were conducted to evaluate the mediation effects of mindfulness traits in the relationship between impulsiveness and anxiety, controlling for age, marital status, educational level, and PA. Impulsiveness was entered in the model as an independent variable, mindfulness trait as a mediator, and each factor of anxiety (cognitive, somatic, and self-efficacy) were evaluated as dependent variables in the three models separately. Indirect effects of impulsivity on each anxiety factor through mindfulness trait were found. 

**Figure 1:**
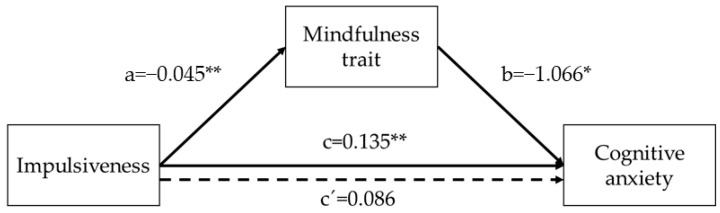
Graphical representation of the mediation effect of mindfulness in the association between impulsiveness and cognitive anxiety. The numerical values correspond to the unstandardized regression coefficients. Dashed line represents the mediated association. ** *p* < 0.01; * *p* < 0.05.

First, impulsivity predicted cognitive anxiety (*B* = 0.135, *SE* = 0.039, *p* = 0.0008) and mindfulness trait (*B* = −0.045, *SE* = 0.005, *p* = 0.0000). Regarding the mediator variables, mindfulness trait predicted cognitive anxiety (*B* = −1.066, *SE* = 0.478, *p* = 0.0269). The analysis of the indirect effect of impulsivity on cognitive anxiety, through the mindfulness trait effect, showed a significant mediation (indirect effect = 0.0487, bias-corrected 95% Confidence Interval for the indirect effect: lower level = 0.0003, upper level = 0.1032). When mindfulness trait was introduced in the model as a mediator, the relationship between impulsivity and cognitive anxiety did not show a statistically significant result (*B* = 0.086, *SE* = 0.045, *p* = 0.0562), suggesting that mindfulness trait has a full mediating effect on that association. Overall, the model (*F*(6,227) = 4.3138, *p* = 0.0004) predicted 10% of the variance in cognitive anxiety in the participants (Figure 1).

**Figure 2:**
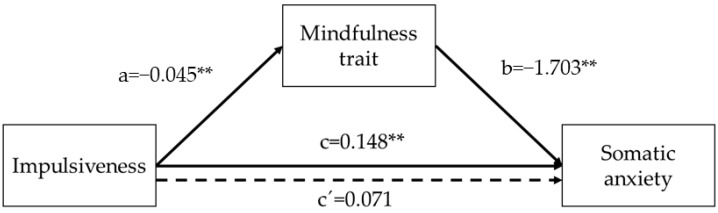
Graphical representation of the mediation effect of mindfulness in the association between impulsiveness and somatic anxiety. The numerical values correspond to the unstandardized regression coefficients. Dashed line represents the mediated association. ** *p* < 0.01.

Regarding the second factor of anxiety, impulsivity predicted somatic anxiety (*B* = 0.148, *SE* = 0.040, *p* = 0.0003) and mindfulness trait (*B* = −0.045, *SE* = 0.005, *p* = 0.0000). Regarding the mediator variables, mindfulness trait predicted somatic anxiety (*B* = −1.703, *SE* = 0.484, *p* = 0.0005). The analysis of the indirect effect of impulsivity on somatic anxiety, through the mindfulness trait effect, showed a significant mediation (indirect effect = 0.0773, bias-corrected 95% Confidence Interval for the indirect effect: lower level = 0.0304, upper level = 0.1286). When mindfulness trait was introduced in the model as a mediator, the relationship between impulsivity and somatic anxiety did not show a statistically significant result (*B* = 0.071, *SE* = 0.045, *p* = 0.1187), suggesting that mindfulness trait has a full mediating effect in that association. Overall, the model (*F* (6,227) = 4.7957, *p* = 0.0001) predicted 11% of the variance in somatic anxiety in the participants (Figure 2).

**Figure 3:**
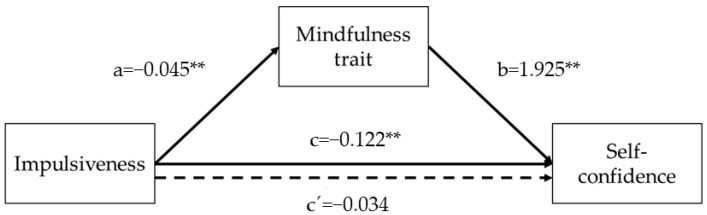
Graphical representation of the mediation effect of mindfulness in the association between impulsiveness and self-confidence. The numerical values correspond to the unstandardized regression coefficients. Dashed line represents the mediated association. ** *p* < 0.01.

Finally, impulsivity predicted self-efficacy (*B* = −0.122, *SE* = 0.034, *p* = 0.0005) and mindfulness trait (*B* = −0.045, *SE* = 0.005, *p* = 0.0000). Regarding the mediator variables, mindfulness trait predicted self-efficacy (*B* = 1.925, *SE* = 0.405, *p* = 0.0000). The analysis of the indirect effect of impulsivity on self-efficacy, through the mindfulness trait effect, showed a significant mediation (indirect effect = −0.0882, bias-corrected 95% Confidence Interval for the indirect effect: lower level = −0.1317, upper level = −0.0498). When mindfulness trait was introduced in the model as a mediator, the relationship between impulsivity and self-efficacy did not show a statistically significant result (*B* = −0.034, *SE* = 0.038, *p* = 0.3682), suggesting that mindfulness trait has a full mediating effect in that association. Overall, the model (*F*(6,227) = 7.6849, *p* = 0.0000) predicted 17% of the variance in self-efficacy in the participants (Figure 3).

## 4. Discussion 

The aims of this study were to determine differences in anxiety, mindfulness, and impulsiveness based on PA levels; to identify the predictive value of sociodemographic and PA, impulsivity, and mindfulness on anxiety factors; and to analyze the possible mediating effects of mindfulness on the relationship between impulsivity and competitive anxiety.

Regarding levels of PA and MET according to sports or type of athletes, the results of the present study have been very similar to those obtained in previous research, both from a national and international perspective [1,81,82,83]. Although the estimation of METs for the different types of sports depends on individual variability in relation to the level of physical condition, dexterity, coordination, efficiency, environmental conditions, intensity, or nature of the effort [81,84], female elite athletes report higher levels of PA. In this study, 30.5% were elite athletes who reported a total of 561 min/day. The team players showed significantly higher levels of METs per day for vigorous PA and MVPA, in addition to lower levels of light PA than the individual sports players. In general, the highest levels of vigorous PA occur in competitive sports athletes [11,84,85]. Although our study did not find statistically significant differences between PA levels, previous studies have found that higher levels were related to a possible decrease of psychological problems, such as anxiety, in people who play sports [66,68]. We found that some studies reported differences between an individual or team sports regarding problems such as anxiety and depression [13] although no significant differences have been found in others [23]. In general, in groups of athletes who practiced vigorous PA, relationships have been found between anxiety in competition and impulsivity [24,34,86]. Specifically, female athletes obtained higher levels of anxiety and may be more prone to suffer psychological problems and greater impulsivity [4,41,60,69,87]. In this sense, our study found significant differences between the variables of attentional impulsiveness and motor impulsiveness with the sample’s PA levels. In addition, the results indicated significant differences between light and moderate PA or vigorous PA in its relation to mindfulness, with higher levels of mindfulness being reported in athletes with higher PA levels [60,88] with the practice of competition related to fewer impulsive responses [34]. In this sense, although there were no differences in anxiety levels based on the intensity of the PA, it has been found that those females who practice light PA, were more impulsive and had a lower mindfulness trait. Secondly, this study identifies the predictive value of sociodemographic factors with PA, impulsivity, and mindfulness in relation to anxiety. Our results seem to show an association between PA levels and self-efficacy anxiety, but it was not significant. These data were supported by studies that found that adolescents [89] and women who practice PA [90,91] had low levels of anxiety. Furthermore, the intensity of PA plays an important role between anxiety and PA levels. The findings showed that athletes who performed vigorous PA presented high levels of self-efficacy anxiety. Contrary to us, Campillo et al. [92] found that moderate and/or vigorous PA were related to less anxiety. Athletes with high anxiety scores tend to manifest behaviors during sports matches that indicate a lack of self-control and a greater tendency towards impulsivity [20,41]. Impulsiveness needs the presence of high concentration to have good self-regulation during the competition [1]. Research studies with elite athletes pointed out that higher levels of mindfulness were associated with fewer negative thoughts, anxiety, and stress in sports situations [12,50,57,58,87]. A significant result of this study was that impulsivity seems to be associated with cognitive anxiety, mindfulness being a mediator between those. When analyzing the possible mediating effects of mindfulness in the relationship between impulsivity and anxiety, our study’s results indicate that the variable mindfulness has a protective effect on anxiety states, inhibiting the effects of impulsivity. These findings were consistent with different studies that support that mindfulness is a protective factor of stress and competitive anxiety in its three components, cognitive, somatic, and self-confidence in sports [93], decreasing the probability of impulsive behaviors and emotion regulation [33,87].

As proposed by some authors, interventions oriented to enhance mindfulness traits could be employed to prevent the negative consequences of impulsiveness on internalizing symptoms such as anxiety [48]. However, to our knowledge, little is known about the possible effects of mindfulness buffering the negative effects of impulsiveness on anxiety in female athletes. Previous studies have found that dispositional mindfulness could refrain maladaptive impulsive behaviors [94,95]. In this regard, it has been demonstrated that the ability to be aware of the present moment and one’s own experience could help individuals to detect their impulsive tendencies, and hence, prevent and regulate them in an effective manner [94]. In athletes, dispositional mindfulness was negatively correlated to impulsiveness [96]. In this sense, in a sports context, the development of behaviors based on mindfulness could be especially relevant, as competitions are stressful situations in which impulsiveness could lead to maladaptive behaviors with negative consequences for both the team and the competition results. Based on path analyses, it has been demonstrated that athletes higher in mindfulness trait develop adaptive emotional regulation strategies to cope with negative emotions [97]. However, it is necessary to know if these positive abilities derived from dispositional mindfulness could prevent negative consequences in highly impulsivity athletes. Considering the close association between emotion regulation difficulties and impulsiveness [98], especially emotional difficulties that were improved by mindfulness training, such as poor emotional clarity, it is necessary to analyze the mediation effect of dispositional mindfulness in the relationship between impulsiveness and anxiety. The obtained results in this regard would reinforce the idea of establishing psychological intervention programs to enhance mindfulness traits in athletes. 

In fact, mindfulness-based strategies have been used in athletes to decrease the effects of competitive anxiety [59,60,87,93] as well as to reduce impulsivity [4,78]. In coherence with these, other studies correlated higher levels of mindfulness with higher levels of PA in female athletes [33,60,88]. For athletes, the literature reports higher levels of mindfulness with higher levels of PA [60,88]. These data coincide with our study: athletes in the vigorous PA group reported the highest levels of mindfulness, in comparison to the moderate or light PA group. Even so, more studies are required of samples of female athletes [88]. For that reason, it seems that mindfulness interventions could be effective strategies to decrease competitive anxiety in its three dimensions, especially in female athletes with high impulsivity levels. 

Specifically, in elite athletes, various studies report that female athletes with high levels of competitive anxiety have a higher prevalence of pre-competitive anxiety [2,41,69], meditation and mindfulness being elements that correlate with better sports performance [31,34,88]. In this sense, different studies with athletes have obtained satisfactory results when using mindfulness-based strategies to reduce the effects of competitive anxiety [59,60,87,93], decrease impulsivity [4,78], and self-control and emotion regulation [33,87,93]. The ability to manage anxiety and impulsive responses through mindfulness could be beneficial against impulsive behavior, control anxiety, and, in turn, improve performance in elite and university female athletes.

## 5. Conclusions

Impulsivity was associated with higher anxiety levels on its different components in elite athletes; however, anxiety was not significantly related to the different PA levels. In this relationship, mindfulness seems to be a mediator between impulsivity and anxiety, and greater mindfulness levels were also reported in athletes who performed vigorous PA. In this sense, the development of mindfulness-based strategies and methods could promote the control of impulsivity and reduce the symptoms of anxiety, thereby improving performance in elite and university female athletes. 

Therefore, the importance that these results can have for coaches, technicians, and all professionals involved in the performance, psychological responses, and health of female athletes is worth highlighting. The development of mindfulness-based strategies and the analysis of their effectiveness in reducing competitive anxiety is highly relevant, especially in elite female athletes with high levels of impulsivity since they seem to be at the greatest risk of suffering from high levels of anxiety.

Although the present study represents significant progress in the analysis of competitive anxiety in female athletes, as well as of risk and protective factors in terms of its development, it has some limitations. First, despite the small sample size, it is a representative sample of a specific group of female athletes, since to our knowledge it is one of the few studies conducted with elite and university female athletes. In addition, the variability of the sample of athletes in different sports enables for generalizing the results obtained. Furthermore, because it is a cross-sectional study, a cause-effect relationship cannot be established in the results obtained. Finally, the questionnaires used are self-report, entailing certain limitations in the participants’ responses, such as the aspects of social desirability or subjectivity of the responses. Future research could be carried out in other sporting events, reporting new data, and comparing them during competitions, according to sports categories and modalities to enable a better generalization of the results, with the aim of analyzing the protective and mediating effect of mindfulness in relation to impulsive responses and anxiety in female athletes in different contexts of competitive sports or of vigorous PA levels.

## Figures and Tables

**Table 1:** Descriptive characteristics of the study sample.

Outcomes	*n* = 242 Mean ± SD
Age, y	22.1 ± 5.2
Height, cm	167.8 ± 7.2
Weight, Kg	62.0 ± 7.9
BMI, Kg/m2	22.0 ± 2.3
Current smoker (%)	24 (9.9%)
Educational levels	
Primary	7 (2.9%)
Secondary	41 (16.9%)
University	194 (80.2%)
Urbanization	
<10,000 inhabitants	12 (5.0)
10,000–50,000 inhabitants	33 (13.7)
>50,000 inhabitants	196 (81.3)
Physical activity level (METs min/day)	
Light PA	86.1 ± 136.2
Moderate PA	114.4 ± 159.8
MVPA	482.4 ± 381.1
Vigorous PA	370.1 ± 336.3
Total PA	561.0 ± 431.8
Sitting time (min/day)	276.9 ± 187.2
Type of sport	
Individual	64 (26.8%)
Team	175 (73.2%)
Sport category	
Elite athletes	73 (30.5%)
University athletes	149 (62.3%)
Regional athletes	17 (7.1%)

Data are expressed as mean ± standard deviation or number of participants (percentages). BMI: Body Mass Index; METs: Metabolic Equivalent of Tasks; PA: Physical Activity; MVPA: Moderate to Vigorous Physical Activity.

**Table 2:** Anxiety, impulsiveness, and mindfulness of the study sample.

Variables	M ± SD
Competitive State Anxiety Inventory	
Somatic anxiety	19.4 ± 5.7
Cognitive anxiety	23.4 ± 5.6
Self-confident Anxiety	24.3 ± 4.9
Barrat Impulsiveness Scale	
Total Impulsiveness	67.2 ± 9.2
Attentional Impulsiveness	19.8 ± 2.9
Motor Impulsiveness	22.9 ± 4.5
Non-planning Impulsiveness	24.6 ± 4.1
Total Mindfulness	4.1 ± 0.9

**Table 3:** Comparisons between PA levels with anxiety, mindfulness, and impulsivity.

Outcomes	Light-Moderate (METS min/Day) (*n* = 113, 46.7%)	Vigorous (METS min/Day) (*n* = 129, 53.3%)	*p* Value	Cohen’s d
Sitting time (min/day)	268.01 ± 195.68	284.60 ± 179.90	0.493	0.088
Anxiety				
Somatic anxiety	19.56 ± 2.7	19.95 ± 3.05	0.209	0.135
Cognitive anxiety	23.94 ± 5.45	22.94 ± 5.69	0.166	0.179
Self-confident anxiety	23.63 ± 4.70	24.85 ± 4.96	0.052	0.252
Total Impulsiveness	69.42 ± 9.02	65.34 ± 8.94	0.001	0.454
Attentional Impulsiveness	20.22 ± 2.95	19.50 ± 2.98	0.034	0.242
Motor Impulsiveness	24.16 ± 4.37	21.80 ± 4.40	0.001	0.538
Non-planning Impulsiveness	25.03 ± 4.50	24.16 ± 3.73	0.107	0.210
Total Mindfulness	3.91 ± 0.86	4.23 ± 0.83	0.004	0.378

Data are expressed as mean ± standard deviation. Differences were run according to normality (Kruskal–Wallis and one-way ANOVA for non-normal and normal distributed variables, respectively). Significant differences between groups are in bold (*p* < 0.05).

**Table 4:** Regression analyses of sociodemographic and physical activity, impulsivity, and mindfulness trait variables as predictors of anxiety factors.

Variable	Cognitive Anxiety					Somatic Anxiety					Self-Efficacy				
	Model 1					Model 1					Model 1				
	B	SE	β	*p*	95% CI	B	SE	β	*p*	95% CI	B	SE	β	*p*	95% CI
Age	−0.115	0.072	−0.109	0.112	[−0.256, 0.027]	−0.033	0.074	−0.031	0.655	[−0.179, 0.113]	0.086	0.063	0.093	0.177	[−0.039, 0.210]
Marital status	−1.117	0.738	−0.102	0.131	[−2.571, 0.336]	−0.234	0.760	−0.021	0.759	[−1.732, 1.264]	−0.551	0.647	−0.058	0.396	[−1.826, 0.725]
Educational level	−0.802	0.746	−0.071	0.283	[−2.272, 0.667]	0.434	0.768	0.038	0.573	[−1.080, 1.947]	−0.918	0.655	−0.092	0.162	[−2.208, 0.373]
PA levels	−0.610	0.727	−0.055	0.402	[−2.044, 0.823]	−0.892	0.749	−0.079	0.235	[−2.367, 0.584]	1.323	0.641	0.136	0.040	[0.061, 2.585]
	*F*(4,233) = 2.154, *p* = 0.019 *R*^2^ = 0.079					*F*(4,233) = 0.569, *p* = 0.686, *R*^2^ = 0.007					*F*(4,233) = 2.186, *p* = 0.071, *R*^2^ = 0.020				
	**Model 2**					**Model 2**					**Model 2**				
	**B**	**SE**	**β**	***p***	**95% CI**	**B**	**SE**	**β**	***p***	**95% CI**	**B**	**SE**	**β**	***p***	**95% CI**
Age	−0.73	0.071	−0.070	0.305	[−0.214, 0.067]	0.011	0.073	0.011	0.876	[−0.133, 0.156]	0.048	0.062	0.052	0.441	[−0.075, 0.171]
Marital status	−1.321	0.724	−0.121	0.069	[−2.747, 0.106]	−0.455	0.743	−0.041	0.541	[−1.919, 1.009]	−0.365	0.634	0.038	0.565	[−1.615. 0.884]
Educational level	−0.870	0.729	−0.077	0.234	[−1.539, 1.324]	0.358	0.749	0.031	0.633	[−1.117, 1.834]	−0.863	0.639	−0.087	0.179	[−2.122, 0.397]
PA levels	−0.107	0.727	−0.010	0.883	[−1.539, 1.324]	−0.338	0.745	−0.030	0.650	[−1.807, 1.130]	0.870	0.638	0.089	0.174	[−0.388, 2.127]
Impulsivity	0.135	0.040	0.224	0.001	[0.057, 0.214]	0.149	0.041	0.242	0.000	[0.068, 0.229]	−0.123	0.035	−0.232	0.001	[−0.191, −0.054]
	*F*(5,233) = 4.113, *p* = 0.001, *R*^2^ = 0.063, ∆*R*^2^ = 0.046, *p* = 0.001					*F*(5,233) = 3.125, *p* = 0.009, *R*^2^ = 0.044, ∆*R*^2^ = 0.054, *p* = 0.000					*F*(5,233) = 4.310, *p* = 0.001, *R*^2^ = 0.067, ∆*R*^2^ = 0.050, *p* = 0.001				
	**Model 3**					**Model 3**					**Model 3**				
	**B**	**SE**	**β**	***p***	**95% CI**	**B**	**SE**	**β**	***p***	**95% CI**	**B**	**SE**	**β**	***p***	**95% CI**
Age	−0.90	0.071	−0.086	0.207	[−0.230, 0.050]	−0.016	0.072	−0.015	0.826	[−0.158, 0.126]	0.078	0.060	0.084	0.196	[−0.041, 0.196]
Marital status	−1.300	0.718	−0.119	0.071	[−2.579, 0.309]	−0.418	0.725	−0.038	0.565	[−1.847, 1.012]	−0.405	0.606	−0.042	0.505	[−1.599, 0.789]
Educational level	−1.135	0.733	−0.100	0.123	[−2.579, 0.309]	−0.071	0.741	−0.006	0.923	[−1.531, 1.388]	−0.372	0.620	−0.037	0.549	[−1.593, 0.849]
PA levels	0.113	0.727	0.010	0.877	[−1.320, 1.546]	0.004	0.734	0.000	0.996	[−1.442, 1.450]	0.457	0.616	0.047	0.459	[−0.757, 1.671]
Impulsivity	0.087	0.045	0.144	0.056	[−0.002, 0.176]	0.071	0.046	0.116	0.119	[−0.018, 0.161]	−0.034	0.038	−0.065	0.368	[−0.110, 0.041]
Mindfulness trait	−1.066	0.479	−0.166	0.027	[−2.010, −0.123]	−1.704	0.484	−0.260	0.001	[−2.658, −0.749]	1.925	0.406	0.641	0.000	[1.126, 2.725]
	*F*(6,233) = 4.314, *p* = 0.0001, *R*^2^ = 0.079, ∆*R*^2^ = 0.020, *p* = 0.027					*F*(6,233) = 4.796, *p* = 0.000, *R*^2^ = 0.089, ∆*R*^2^ = 0.048, *p* = 0.001					*F*(6,233) = 7.685, *p* = 0.000, *R*^2^ = 0.147, ∆*R*^2^ = 0.083, *p* = 0.000				

PA: Physical activity. Significant differences were set up at the level of (*p* < 0.05).

## Data Availability

The data are not publicly available due to reasons concerning privacy of the subjects and since it belongs to an ongoing project.
